# Reversible Verbal Memory Integration Deficits in Obstructive Sleep Apnoea

**DOI:** 10.5334/pb.1035

**Published:** 2021-03-25

**Authors:** Oumaïma Benkirane, Daniel Neu, Rémy Schmitz, Hedwige Dehon, Olivier Mairesse, Philippe Peigneux

**Affiliations:** 1 UR2NF – Neuropsychology and Functional Neuroimaging Research Group at CRCN – Center for Research in Cognition and Neurosciences, Université Libre de Bruxelles (ULB) and UNI – ULB Neurosciences Institute, Brussels, Belgium; 2 Brugmann University Hospital, Sleep Laboratory & Unit for Chronobiology U78, Université Libre de Bruxelles (ULB), Brussels, Belgium; 3 UNI, ULB Neurosciences Institute, Research unit ULB312 (Faculty of Medicine) and ULB388 (Faculty of Motor Sciences), Université Libre de Bruxelles (U.L.B.), Brussels, Belgium; 4 Center for the Study of Sleep Disorders, DELTA Hospital, Neuroscience Pole, CHIREC, Brussels, Belgium; 5 PsyNCog – Psychology & Neuroscience of Cognition, Université de Liège, BE; 6 Royal Military Academy (R.M.A.), Department LIFE (Physiology and Performance), Brussels, Belgium; 7 Department EXTO, Vrije Universiteit Brussel (V.U.B.), Brussels, Belgium

**Keywords:** OSA, Positive Airway Pressure, Follow-up, Verbal Memory, Episodic Memory, False Memory

## Abstract

When presented with novel but semantically related elements after learning verbal material, healthy participants tend to endorse these items as previously learned. This reflects the normal integration and association of novel verbal information into long-term memory. How obstructive sleep apnoea (OSA) negatively impacts verbal memory performance, and whether deficits are reversible following positive airway pressure (PAP) treatment, remain elusive. We investigated immediate and delayed OSA- and PAP treatment-related effects on verbal memory integration, using a false memory paradigm. Twenty-three patients with OSA learned lists of words semantically related to target non-presented words (1) at baseline after a polysomnography diagnosis night, (2) after a consecutive polysomnography night under PAP titration, and (3) after three months of compliant PAP treatment. At each session, participants learned 10 different lists of words, each list comprising 15 semantically related items. They had then to recognize 15 minutes later (after an intermediate vigilance task) previously learned words within a list including studied words (learned), unstudied but semantically related items (lures), and non-related unstudied items (controls). Sleep quality and fatigue questionnaires, and psychomotor vigilance tests (PVT) were administered at each session. PAP treatment led to OSA remission and improvement in objective and subjective sleep quality. Crucially, recognition of learned and lure words increased after the first night under treatment and remained stable three months later, suggesting successful memory integration and restoration of semantic processes. No treatment-related outcome was found on PVT performance. OSA exerts a detrimental but PAP-reversible effect on verbal learning and semantic memory integration mechanisms underlying the acquisition of novel memory representations.

## Introduction

Obstructive sleep apnoea (OSA) negatively impacts various domains including quality of life, productivity, alertness, activity levels, social interactions and sexual health (Weaver & George, 2011). Clinically, OSA features recurrent upper airway collapses (i.e. obstructive hypopneas and apnoeas) with intermittent hypoxemia, leading to frequent arousals, potential slow-wave sleep reduction, and/or rapid eye movement sleep depletion, and increased light sleep proportions (Barner et al., 2016). OSA-related sleep disruptions also impact cognitive functions (Luz et al., 2016; Wallace & Bucks, 2013). Verbal memory deficits, in particular, have been reported in OSA (Kloepfer, 2009), other reports additionally suggesting visuospatial episodic memory (Wallace & Bucks, 2013) and implicit sequence learning (Csábi et al., 2014) deficits. Conversely, there is limited evidence for memory improvement (Zimmerman et al., 2006; Sforza & Roche, 2012) after continuous positive airway pressure (CPAP) treatment. This treatment operates as a pneumatic splint that sustains the upper airway opening by delivering constant positive air pressure through a mask (Deacon et al., 2016). Its use has been proven to significantly lessen the apnea-hypopnea index (AHI), an average joined count of respiratory events (apneas and hypopneas) developing per hour of sleep (Pavwoski & Shelgikar, 2017). It is currently considered the first-line, gold standard treatment for moderate (15 < AHI < 30) and severe (AHI > 30) OSA (Rotenberg et al., 2016) and can also be adapted to mild but symptomatic cases (Buchanan & Grunstein, 2011). CPAP has been shown to overcome sleep-related symptoms and increase quality of life (Laratta et al., 2017). Cognitive functions, e.g., visual (Lin et al., 2015) and short-term memory (Canessa et al., 2011), seem to be partially restored by standard OSA treatment such as positive airway pressure (PAP), but the reversibility of OSA-related deficits in verbal memory following PAP treatment needs to be further investigated.

Impaired sleep quality is detrimental to verbal memory, both at encoding after disrupted sleep or during memory consolidation as tested after post-learning sleep (Rasch, & Born, 2013). In healthy subjects, prior sleep quality is associated with verbal memory performance (Cipolli, Mazzetti, & Plazzi, 2013). In chronic sleep disorders such as OSA, sleep alterations may be detrimental to memory encoding (for a review, see Cipolli, Mazzetti, & Plazzi, 2013). Besides, it is possible to delineate altered verbal memory components by distinguishing between recalling a memorized item and its shallower integration into semantic memory. According to the dual-process Fuzzy-Trace Theory (FTT) model (Brainerd & Reyna, 1998), word recognition involves the simultaneous memorisation of the general idea to which it refers, in other terms its “deep” meaning (Gist memory) but also the specificity of this word (Verbatim or item-specific memory). Therefore, the representation of the word is not memorized as a single entity. Rather, there is parallel encoding with separate storage of Gist and Verbatim traces, that could be separately activated. In this framework, the memory illusion eventually leading to a false memory may result either from the recovery of the representation of the general idea of the words in the learning list rather than from their specifics, or from the recovery of an erroneous Verbatim trace. Exact memory would be subtended by the recovery of a correct Verbatim trace. The formation of false memories has been mainly studied using the DRM paradigm (Deese, 1959; Roediger & McDermott, 1995). In the DRM procedure, participants are first asked to memorize lists of words (e.g., a list with the words “trunk”, “branch”, “leaf”, “wood”, “roots”, “fir oak”, “strain”, “lumberjack”…). They are not informed that all words in a learning list are actually semantically linked to the same semantic field (“tree” in the present example) which is not presented in the list of words to remember. In this framework, considering that a new word (e.g., “trunk”) is not only memorized as a specific item but also integrated into the related existing semantic networks (e.g., “tree”), this normal integrative mechanism may paradoxically lead to a so-called false memory effect. Indeed, when presented with semantically related but unstudied elements (e.g., the word “tree”), participants will tend to erroneously recognize the new item as previously learned (Arndt, 2012), besides correctly recognizing the studied ones. Critical lures and words presented within the studied list were shown recognized at a similar rate (e.g., 55%; Roediger & McDermott, 1995), and up to 70% of semantically associated words may be falsely recognized as previously learned (Payne et al., 1996; De Brigard, 2014). Consequently, it can be argued that the production of semantically related (but false) memories reflect normal integration mechanisms in human memory (Schacter, Guerin, & St Jacques, 2011; Pardilla-Delgado & Payne, 2017).

In this context, patients with OSA were shown to exhibit poorer discrimination than healthy controls when facing a forced-choice recognition between previously learned words (e.g. “trunk”) and the critical lure (e.g., “tree”), but to achieve similar performance levels when memory performance could be based on Gist information (e.g., “trunk” vs “piano”; Sarhane et al., 2014). It suggests that patients with OSA mostly stored the general idea (gist) of the learned information but were less efficient with the item-specific (verbatim) memory. Memory deficits in OSA appear to be most important when patients must recollect specific details of the learning episode (e.g., item’s specific context or source; Sarhane & Daurat, 2017). Consequently, recollection deficit in OSA patients would eventually lead to higher and easier acceptance in a recognition task of novel items when they are conceptually related to previously learned items. However, this interpretation might be disputed based on prior findings. Indeed, it was shown that the susceptibility to memory illusions (as investigated with the DRM paradigm) tends to increase with age (Dehon, 2006; Dehon & Brédart, 2004; Butler, 2004). Accordingly, a meta-analysis of 232 studies reported better performance in recognition tasks in young than in older adults, the latter having a propensity bias for more liberal answering eventually leading to an increment in false alarms (Fraundorf et al., 2019; Sejunaite et al., 2019). The facts that verbal memory difficulties in patients with OSA can be seen as equivalent to those experienced by healthy controls 10 years older (Twigg et al., 2010) and/or that age-related heterogeneity is higher for item-specific than gist-based memory tasks (Brainerd & Reyna 2005, in Latour, Latour, & Brainerd, 2014) may explain the reported dissociation between item-specific and gist memory in patients with OSA (Sarhane et al., 2014). Additionally, increased susceptibility of patients with OSA to false recognition may stem from a limited availability of attentional resources eventually disrupting recovery processes (Sarhane & Daurat, 2019). This is in line with prior reports showing that young adults achieve similar performance levels than older adults when cognitive resources are reduced at encoding (Dehon 2006),

In this framework, the present study aimed at investigating immediate and delayed CPAP treatment-related effects on verbal memory integration in OSA. Participants with OSA were tested using a controlled semantic memory paradigm aimed at inducing false memories (DRM; Deese, 1959a; Roediger & McDermott, 1995), first in an untreated condition then immediately after PAP initiation, and after three months of PAP compliant use. We expected PAP treatment to increase overall episodic memory performance, both for the recall of learned (exact) words and the integration of non-learned but semantically related words (i.e., “false” memories).

## Methods

### Participants

23 French-speaking male participants (55.39 ± 8.92 years) gave written informed consent to participate in this study approved by the Brugmann Hospital Ethics committee (CE 2016/86). Inclusion criteria were PSG-based OSA diagnosis and an obstructive apnoea-hypopnea index (OAHI) > 15/hour during sleep. From this cohort, 12 patients (55.25 ± 10.17 years) were tested three months later (average PAP compliance 5.1h/24h ± 0.37 during the three months period). At enrolment in the protocol, exclusion criteria were PAP treatment prior to the study, neurological or psychiatric comorbid conditions, history of opioid treatment or current benzodiazepine intake. Participants were also asked to avoid stimulating and/or alcoholic drinks the day before as well as during the experimental days. For the 12 participants tested three months later, the inclusion criterion was to sleep at least five days a week for at least four hours per night under fixed air pressure administration from the introduction of the PAP treatment to the follow-up session.

### Design and Procedure

#### Pre-testing questionnaires and tasks

Upon admission to the Sleep Laboratory, before the polysomnography (PSG) diagnosis night, participants were administered a battery of psychometric instruments, including the Morningness-Eveningness questionnaire (MEQ, circadian typology; Ogiska, 2011), the Pittsburgh Sleep Quality Index (PSQI, sleep quality of the previous month; Buysse et al., 1989), the fatigue severity scale (FSS, perceived impact of fatigue on the previous month; Krupp et al., 1989), the Brugmann Fatigue Scale (BFS, the behavioral impact of physical and mental fatigue; Mairesse et al., 2017), the Epworth Sleepiness Scale (ESS, sleep propensity in specific situations; Johns, 1991) and the hospital anxiety and depression scale (HADRS; Zigmond & Snaith, 1983). To control for verbal abilities, phonological and categorical fluency tasks (Cardebat et al., 1990) were administered: participants had to produce as many different words as possible within one minute, either starting with the same phoneme (P; phonological) or belonging to the same semantic category (animals; categorical). Additionally, they completed the Mill-Hill Vocabulary Scale Part B (Deltour 1993), in which a synonym must be selected out of 6 propositions for 33 different words of increasing complexity. Cut-offs were not used for inclusion-exclusion of the participants, these tests aiming for a clinical characterization. As the experimental task was verbal (see below), screening for participants’ French fluency was conducted informally by the main experimenter as an interview with each participant when explaining the modalities of the experiment.

#### Testing procedure

In the morning following their first night in the sleep unit and after confirmation of the OSA diagnosis, patients completed the St-Mary’s Sleep Questionnaire (Ellis et al., 1981) subjectively assessing the quality of the preceding night. They then underwent a first experimental session in the sleep laboratory. During this session, they were administered (1) the learning phase of the DRM (see below; Deese, 1959a; Roediger & McDermott, 1995), (2) a 10-minute version of the psychomotor vigilance task (PVT; Basner et al., 2015) and (3) the test phase of the DRM. Sleepiness (Babkoff, Caspy & Mikulincer, 1991), fatigue (Lee et al., 1991), and mood (Nyenhuis et al., 1997) scales were administered before and after each phase.

During the subsequent PSG night in the sleep laboratory, CPAP was initiated, and adequate air pressure titrated. A second experimental session (identical procedure) was held the following morning. Finally, a third experimental session (identical procedure) was held three months later (without PSG recording).

#### DRM false memory task

The DRM (Deese, 1959a; Roediger & McDermott, 1995) is a paradigm aimed at inducing false memories using a controlled material. In the learning phase of each experimental session, 10 separate lists of words were orally presented to the participants who were asked to carefully listen and memorize. The words were uttered by a recorded female voice at a one-word-per-second frequency, with at least a 10-second break between every list. Each list included 15 items semantically related to a theme (or lure) word not included in this list (e.g. “trunk”, “branch”, “leaf”, “wood”, “roots”, “fir oak”, “strain”, “lumberjack”… related to the word “tree”). Ten different lists were presented at each session, out of a choice of 30 different lists previously shown to be comparable in terms of saliency and frequency of appearance of the words (Darsaud et al., 2011; Schmitz, Dehon, & Peigneux, 2013). It should be noticed that while the DRM task here is similar to the one used by Darsaud et al. (2011) who aimed at investigating sleep-dependent memory consolidation effects, we focused here on the effect of *prior* sleep quality on verbal memory performance with a limited time interval between the learning and recognition phases. Results will thus not be comparable.

During the test phase following the PVT, participants had to identify the previously studied words within a list of 60 words successively displayed on the computer’s screen. The testing material included 20 studied items, 10 lures (i.e., non-studied but semantically related theme words), and 30 distractors (unrelated, non-studied words). Those distractors englobed 10 distractors matched with lures, and 20 distractors matched with studied items, in terms of gender, grammatical class, number of syllables, frequency, and the absence of semantical association to the studied words or between them (see Dehon & Brédart, 2004, for a detailed presentation of the material). The 20 studied items were the first and the twelfth of each studied list. Prior DRM experiments using a recognition testing modality also presented studied words at precise *n* position of the studied list (e.g., positions 1, 8, and 10 in Roediger & McDermott, 1995) to test for the degree of semantic association effects, the words in a list being ordered from the highest to the lowest semantic association strength with the lure. To the best of our knowledge, similar memory performance was found in all of these studies, suggesting that participants do not take advantage of the correlation between item position and presence in the testing material.

When a word was recognized as studied, participants were asked to specify whether their recognition was based on a clear memory recollection of the studied word (Remember), a feeling of familiarity about the word (Know), or if their response was at random (Guess) (Gardiner, Ramponi, & Richardson-Klavehn, 1998). The experiment was implemented using Cogent 2000 (*http://www.vislab.ucl.ac.uk*) running on Matlab R2014a.

#### Sleepiness, fatigue, mood, and alertness

At the beginning of each experimental session, three visual analog scales (VAS) were administered. The first assessed the perceived level of sleepiness (VAS-S; Babkoff, Caspy, & Mikulincer, 1991), the second evaluated subjective fatigue (VAS-F; Lee et al., 1991), and the third assessed subjective mood (VAS-M; Nyenhuis et al., 1997). At each session, participants completed the scales three times (after DRM learning, after PVT, and after DRM recognition).

The 10-min version of the computerized Psychomotor Vigilance Task (PVT; Basner et al., 2015) was also administered. In this task, patients had to press a key as fast as possible as soon as a 1 msec incrementing counter appears on-screen. Stimuli were displayed at intervals ranging randomly from 2, 4, 6, 8, and 10 sec.

#### Polysomnography and PAP

Clinical PSG recordings were conducted using standard guidelines defined by the American Academy of Sleep Medicine (AASM, 2014), including high-resolution infrared videography. PSG recordings included at least Fp2-Ax, C4-Ax, and O2-Ax derivations, two electrooculograms, anterior and bilateral anterior tibial electromyograms. Oral and nasal airflow was recorded by an oro-nasal cannula (Pro-Flow Plus™ Pro-Tech® Mukilteo, WA, USA). Respiratory effort was measured by thoracic and abdominal belts (Pro-Tech® CT2™, Mukilteo, WA, USA). Capillary oxygen saturation was monitored by photo-oximetry (Nonin® Flexi-Form® II 7000A Nonin Medical Inc., Minneapolis, MN, USA et LINOP® Adt Masimo corp. Irvine, CA, USA). All PSG recordings were analyzed on 21” screens with epochs of 30-second polysomnography (Philips Respironics Inc™ Alice6®, Philips Healthcare™, Eindhoven, NL, EU) by qualified technicians unaware of the objectives of the study. PAP-devices were Auto-Continuous PAP (automatic pressure titration) DreamStation® (Philips Respironics Inc™).

### Statistical analyses

Statistical analyses were performed using IBM SPSS 25® (International Business Machines, SPSS™, Armonk, NY, USA). Immediate and sustained treatment effects were investigated using Linear Mixed Models with time as the fixed effects and subjects as the random effects. Models were estimated through Restricted Maximum Likelihood with a First-Order Autoregressive (AR1) covariance structure. Distractor-related responses were subtracted from true and false memory data to obtain induced-memory scores purged from intrusions. Pearson correlations were performed on memory data and sleep parameters before and after PAP-treatment.

## Results

### Population sample characteristics

Demographic variables and scores measured at enrolment are reported ***Table 1***, both for the 23 participants having participated to the first and second experimental sessions (two successive days), and for the subsample of 12 having participated to the third experimental session 3 months later.

**Table 1:** Demographic features of original and follow-up testing samples.


	ORIGINAL SAMPLE (S1–S2)	RANGE S1–S2	FOLLOW–UP SAMPLE (S3)	RANGE S3

Age	55.95 (9.15)	42–73	55.25 (10.17)	42–73

PSQI	8.21 (4.06)	2–16	7.73 (4.92)	2–16

ESS	8.65 (4.65)	3–20	9.17 (4.90)	3–20

FSS	3.76 (1.87)	1,22–7	3.85 (1.98)	1,22–7

Mental BFS	3.55 (2.69)	0–9	1.5 (0.25–3)	0–8

Physical BFS	4.3 (2.66)	0–10	3.5 (2.20)	0–8

Morningness-Eveningness scale	21.5 (8.61)	12–40	20 (7.19)	12–32

Distinctness scale	17.10 (7.21)	6–30	16 (8)	6–30

HADS Total	12.45 (5.79)	3–28	12.08 (6.68)	3–28

HADS Anxiety	6.5 (3.46)	2–15	6.08 (3.61)	2–15

HADS Depression	5.95 (3.66)	1–13	6 (3.98)	1–13

Mill Hill	21.57 (6.82)	4–33	23.58 (5.14)	14–33

Phonological fluency	22.20 (6.98)	13–38	21.08 (7.38)	13–38

Semantical fluency	26.29 (6.51)	15–38	27.25 (6.06)	18–36

Reciprocal reaction time (PVT)	3.03 (.26)	2.48–3.46	3.02 (0.20)	2.71–3.34


*Note:* For the original testing sample (sessions S1 and S2), n = 23 except PSQI n = 21. For the follow-up sample (session S3), n = 12 except PSQI n = 11. PSQI = Pittsburgh Sleep Quality Index (PSQI, sleep quality of the previous month; Buysse et al., 1989); FSS = Fatigue Severity Scale (Krupp et al., 1989). BFS = Brugmann Fatigue Scale (Mairesse et al., 2017). ESS = Epworth Sleepiness Scale (Johns 1991). HADS = Hospital Anxiety and Depression Scale (Zigmond & Snaith, 1983). PVT = Psychomotor Vigilance Test (Basner et al., 2015). Data are given as mean (SD) or median (quartile 1-quartile 3). For the reciprocal reaction time in the PVT, the wilcoxon signed-rank tests testing for differences between S1 and S2 (Z = –1.65, *p* = .1) and between S2 and S3 (–1.73, *p* = .084) were non-significant.

### Subjective sleep quality

Subjective sleep satisfaction (Ellis et al., 1981) improved after the first night of CPAP (n = 23; all *p*’s < .05). Subjective sleep parameters (Ellis et al., 1981) improved in all domains after three months of compliant use (n = 12; all *p*’s > .05; see ***Table 2***).

**Table 2:** Subjective sleep quality for the night before the experimental sessions.


	BASELINE (S1)	CPAP TRIAL (S2)	FOLLOW-UP (S3)	S2–S1	S3–S1

	M (SD =)	M (SD =)	M (SD =)		

Sleep depth	4.09 (.33)	4.63(.35)	6.42 (.47)	Δ = .55; *p* = .18	Δ = 2.33; *p* < .001

Sleep quality	2.78 (.24)	3.25 (.25)	4.97 (.35)	Δ = .47; *p* = .16	Δ = 2.18; *p* < .001

Sleep satisfaction	2.44 (.23)	3.12 (.24)	4.01 (.33)	Δ = .68; *p* = .03	Δ = 1.57; *p* < .001

Nocturnal awakenings	2.78 (.35)	2.70 (.36)	.51 (.48)	Δ = –.09; *p* = .82	Δ = –2.28; *p* < .001

Perceived difficulty of falling asleep	1.83 (.15)	2.04 (.16)	.84 (.21)	Δ = .21; *p* = .26	Δ = –.99; *p* < .001


*Note*: Subjective Sleep quality (Ellis et al., 1981) mean (standard deviation) values for the nights preceding the verbal learning sessions, and between-sessions comparison statistics. Δ: differential score between sessions.

### Polysomnography

All polysomnographic parameters improved from baseline to the first night when PAP treatment was initiated (see ***Table 3***).

**Table 3:** Polysomnographic parameters at Baseline (S1) and CPAP trial (S2) nights.


	BASELINE	CPAP TRIAL	BASELINE VS. CPAP TRIAL NIGHT	DIRECTIONALITY

	M (SD)	M (SD)	F, P	

**TIB**	515.75 (7.47)	510.26 (7.47)	*F*(1,22) = .83 *p* = .37	S1 = S2

**TST**	357.41 (15.93)	346.94 (15.93)	*F*(1,22) = .39 *p* = .54	S1 = S2

**SPT**	424.33 (15.43)	411.74 (15.43)	*F*(1,22) = .74 *p* = .4	S1 = S2

**Sleep efficiency**	68.95 (2.55)	67.7 (2.55)	*F*(1,22) = .20 *p* = .66	S1 = S2

**Total number of Arousals**	241.59 (15.86)	113.68 (15.86)	*F*(1,21) = 57.96 *p* < .01	S1 > S2

**Snore (%)**	36.51 (4.10)	11.26 (4.10)	*F*(1,22) = 33.06 *p* < .01	S1 > S2

**N1 (%)**	18.25 (1.99)	13.97 (1.99)	*F*(1,22) = 4.23 *p* = .05	S1 > S2

**N2 (%)**	52.10 (2.83)	48.33 (2.83)	*F*(1,22) = 1.25 *p* = .28	S1 = S2

**N3 (%)**	17.08 (2.25)	19.99 (2.25)	*F*(1,22) = 1.23 *p* = .28	S1 = S2

**REM (%)**	12.57 (1.19)	17.72 (1.19)	*F*(1,22) = 16.93 *p* < .01	S1 < S2

**AHI (/h)**	39.9 (3.08)	10.14 (3.08)	F(1,22) = 73.53 p < .01	S1 > S2

**AHI REM (/h)**	33.74 (3.30)	6.97 (3.30)	*F*(1,22) = 38.62 *p* < .01	S1 > S2

**AHI NREM (/h)**	40.44 (3.33)	10.95 (3.33)	*F*(1,22) = 63.78 *p* < .01	S1 > S2

**OAHI (/h)**	37.31 (3.13)	8.26 (3.13)	*F*(1,21) = 66.10 *p* < .01	S1 > S2

**RDI (/h)**	45.1 (3.02)	12.19 (3.02)	*F*(1,21) = 99.59 *p* < .01	S1 > S2

**RDI REM (/h)**	37.54 (3.46)	8.39 (3.46)	*F*(1,22) = 37.66 *p* < .01	S1 > S2

**RDI NREM (/h)**	45.60 (3.12)	13.17 (3.12)	*F*(1,22) = 95.93 *p* < .01	S1 > S2

**ODI (/h)**	27.06 (3.53)	7.02 (3.53)	*F*(1,22) = 28.58 *p* < .01	S1 > S2

**Mean Saturation**	27.05 (3.53)	7.02 (3.53)	*F*(1,22) = 28.58 *p* < .01	S1 > S2

**ArI Total**	241.59 (15.89)	113.68 (15.89)	*F*(1,21) = 57.96 *p* < .01	S1 > S2

**ArI respiration**	27.52 (4.2)	9.48 (4.2)	*F*(1,22) = 11.70 *p* = .02	S1 > S2

**ArI Desaturation**	96.87 (10.59)	15.65 (10.59)	*F*(1,22) = 42.76 *p* < .01	S1 > S2


*Note*: TIB = time in bed; TST = total sleep time; SPT = sleep period time; N1(%) = proportion (%) sleep stage N1 on TST; N2(%) = % sleep stage N2; N3(%) = % sleep stage N3; REM (%) = % sleep stage REM; AHI = apnoea and hypopnea index; AHI REM = apnoea and hypopnea index during REM stage; AHI NREM = apnoea and hypopnea index during Non-REM stages; OAHI = obstructive apnoea and hypopnea index; RDI = respiratory disturbance index; RDI REM = respiratory disturbance index during REM stage; RDI NREM = respiratory disturbance index during Non-REM stages; ODI = oxygen desaturation index; ArI total = total arousals index; ArI respiration = respiratory related arousals index; ArI desaturation = desaturation related arousals index. Directionality in the last column indicates the direction of the differences between Baseline and CPAP trial nights, with S1 = S2 meaning no statistically significant differences.

### Sleepiness, fatigue, mood, and alertness

Subjective evaluations (visual analog scales) remained stable throughout the three sessions for sleepiness (sleepiness at the first session = 3.98 ± .5, sleepiness at the second session = 3.80 ± .52, sleepiness at the last session = 2.61 ± .68; *F*(2, 31.82) = 1.65, *p* = .21), fatigue (fatigue at the first session = 4.54 ± .47, fatigue at the second session = 4.16 ± .49, fatigue at the last session = 2.85 ± .65; *F*(2, 32.65) = 2.41, *p* = .11) and mood (mood at the first session = 7.97 ± .5, mood at the second session = 7.82 ± .51, mood at the last session = 6.79 ± .63; *F*(2, 29.09) = 1.81, *p* = .18).

Likewise for the PVT, evaluation of reciprocal reaction time (RRT (Basner et al., 2015)) with Session as the within-subject factor (S1 vs. S2 vs. S3) did not reveal variations in vigilance (RRT at S1 = 3.07 ± .07, RRT at S2 = 3.04 ± .07, RRT at S3 = 3.004 ± .1; *F*(2, 30.20) = .15; *p* = .86).

### DRM false memory task

**Figure 1:**
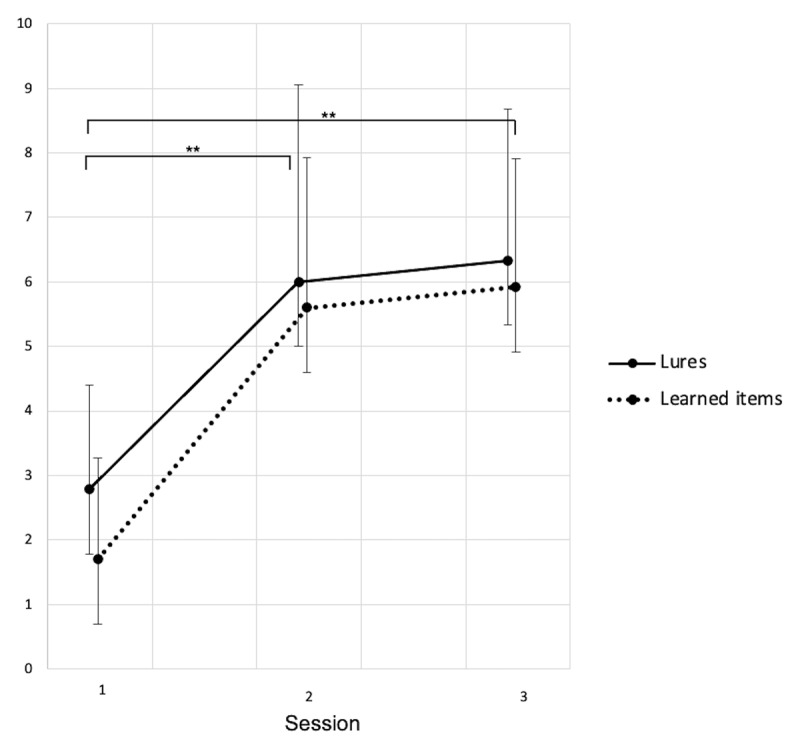
Recognition score (number of words endorsed) for learned words and lures. Session 1: Baseline, Session 2: After CPAP-trial, Session 3: Follow-up. Data shown as mean ± standard deviations. S1–S2: 23 participants. S3: 12 participants. * *p* < .05, ** *p* < .01.

Recognition scores for learned items, computed as the number of correct recognitions minus the false alarms for distractors, was different between sessions (S1: 1.54 ± .40, S2: 5.57 ± .41, S3: 5.68 ± .53; *F*(2, 34.8) = 41.41, *p* < .001; ***Figure 1***). Memory performance improved from baseline to after the night under CPAP (Δ_S2–S1_ = 4.02, *p* < .001; *d* = –1.69), then remained stable after three months (Δ_S3–S1_ = 4.14, *p* < .001, *d* = –2.12; Δ_S3–S2_ = –.12, *p* = .84, *d* = .12).

Recognition scores for lures (i.e., related “Theme” words) computed as the number of endorsed lures minus false alarms for distractors was different between sessions (S1: 2.78 ± .50, S2: 6.03 ± .52, S3: 6.14 ± .66; *F*(2, 35.43) = 18.52, *p* < .001). Lure acceptance increased from baseline to after the CPAP trial (Δ_S2–S1_ = 3.25, *p* < .001, *d* = –1.15), then remained stable after three months (Δ_S3–S1_ = 3.36, *p* < .001, *d* = –1.28; Δ_S3–S2_ = –.11, *p* = .88, *d* = 0).

**Figure 2:**
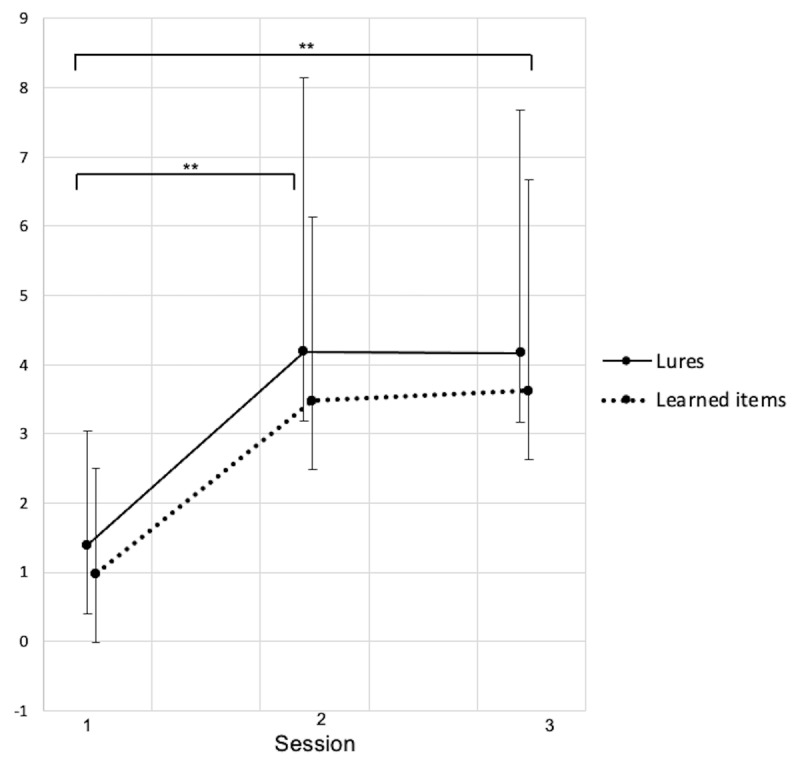
Recognition score (number of words endorsed) for learned words and lures for which the recognition was based on a clear memory of the studied word (Remember). Session 1: Baseline, Session 2: After CPAP-trial, Session 3: Follow-up. Data shown as mean ± standard deviations. S1–S2: 23 participants. S3: 12 participants. * *p* < .05, ** *p* < .01.

**Figure 3:**
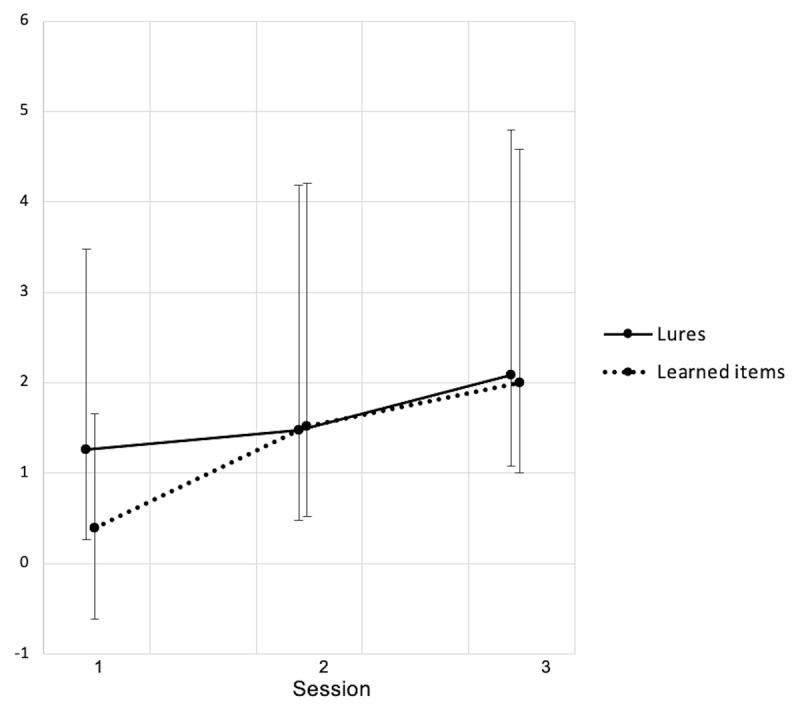
Recognition score (number of words endorsed) for learned words and lures for which the recognition was based on a feeling of familiarity about the word (Know). Session 1: Baseline, Session 2: After CPAP-trial, Session 3: Follow-up. Data shown as mean ± standard deviations. S1– S2: 23 participants. S3: 12 participants. * *p* < .05, ** *p* < .01.

**Figure 4:**
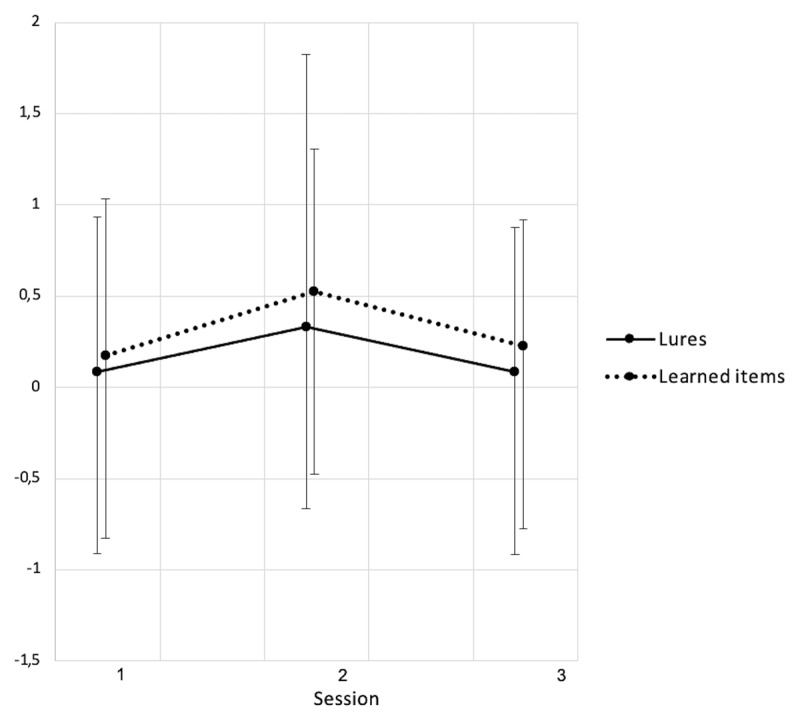
Recognition score (number of words endorsed) for learned words and lures for which the recognition was at random (Guess). Session 1: Baseline, Session 2: After CPAP-trial, Session 3: Follow-up. Data shown as mean ± standard deviations. S1–S2: 23 participants. S3: 12 participants. * *p* < .05, ** *p* < .01.

Additional analyses conducted on participants› confidence in their responses (i.e., Remember, Know or Guess, see ***Figures 2, 3*** and ***4***) showed that Remember responses for targets was different between sessions both for studied word (S1:.98 ± .50, S2: 3.45 ± .51, S3: 3.72 ± .66; *F*(2, 34.86) = 11.05, *p* < .001) and lures (S1: 1.39 ± .63, S2: 4.17 ± .65, S3: 4.54 ± .83; *F*(2, 36.28) = 9.95, *p* < .001). Recognition based on a clear memory of the presented word similarly increased from baseline to after the CPAP trial for studied words (Δ_S2–S1_ = 2.47, *p* < .001, *d* = –1.1) and lures (Δ_S2–S1_ = 2.78, *p* < .001, *d* = –.89), then remained stable at three months both for learned items (Δ_S3–S1_ = 2.74, *p* = .001, *d* = –1.08; Δ_S3–S2_ = .27, *p* = .70, *d* = –.24) and for lures (Δ_S3–S1_ = 3.15, *p* = .002, *d* = –1.13; Δ_S3–S2_ = .37, *p* = .66, *d* = –.39). No changes were found for familiarity-based (Know) or random (Guess) responses (all *p*’s > .05).

At the baseline session, recognition scores for learned items correlated with total sleep time (r = –.56, *p* = .006), sleep efficiency (r = –.5, *p* = .015) and total number of arousals (r = –.47, *p* = .03) for the prior night. When tested at the three-months session, recognition scores for lures correlated with PAP compliance (r = .81, *p* = .015).

No initial (1^st^ and 2^nd^ sessions S1 and S2) differences were found between the 12 participants who completed the 3 sessions of the study (CPAP compliant use) and the 11 who did not in recognition scores for learned items and lures. Similarly, vigilance (reciprocal reaction time) was similar between subgroups (see ***Table 1***).

## Discussion

In the present study, we investigated PAP treatment-related effects on the storage and integration of information into verbal memory following immediate and delayed (three months) treatment schedules in individuals presenting with OSA. Our results evidence reversible OSA-related deficits in verbal memory both for the retrieval of learned items and for semantic generalization (i.e., acceptance of semantically related but non-learned items), with a significant improvement in performance after a single night under PAP treatment, persisting three months later under compliant treatment conditions. These findings are in line with previous studies relating neuropsychological impairment in OSA to sleep fragmentation and intermittent nocturnal hypoxia (Sankri-Tarbichi, 2012), reversible under CPAP treatment (Sforza & Roche, 2012), with a focus on verbal learning and memory abilities.

As compared to the non-treated baseline condition, the number of correctly recognized (i.e. really studied) items significantly increased after one night under PAP-treatment and persisted three months later, suggesting that compliant CPAP treatment reinforced overall episodic memory abilities. Previous reports likewise suggest that verbal memory impairments in OSA patients may be reversible under CPAP treatment (Rosenzweig et al., 2016; Sforza & Roche, 2012). Furthermore, endorsement of semantically related but not learned items (i.e., the lures) also increased after the initiation of PAP treatment and remained stable at three months. Considering these results, we propose that items never memorized but related to the learning lists’ theme (i.e., lures) have been more efficiently linked with associated representations in the pre-existing semantic network after sleep quality was restored by PAP. From another point of view, a prior study interpreted the higher production of false memories in patients with OSA than in healthy controls as indicative of a failure of the episodic component (i.e., temporal and spatial context) of verbal memory (Sarhane et al., 2014). Although production of false memory might indeed stem from episodic, contextual memory deficits (Daurat et al., 2008), it may also, in the DRM context, reflect the normal semantic categorization and generalization processes by which we integrate novel information into pre-existing knowledge stores, which is essential to daily cognitive functioning (Schacter, Guerin, & St Jacques, 2011). In this framework, memory distortions would result from evolutionarily based adaptive cognitive processes (Brainerd & Reyna, 2005; Schacter, 2001). Indeed, associative processes would provide the structure and organization that supports the general efficiency of memory performance, whereas gist-based processes would support the retention of themes and meanings, therewith facilitating generalization and abstraction. Gist-based memory mechanisms may additionally decrease the load of memory storage by forming condensed event records (i.e., the general idea) without the need to maintain many contextual details that might not need to be recalled later on. Remembering information based essentially on conceptual representations of the target event may thus occasionally lead to false memories, as missing details are reconstructed (Schacter, Guerin, & St Jacques, 2011).

Besides, the use of high-order cognitive abilities may allow individuals to set deliberate strategies to avoid the formation of false memories (Gallo, 2006). Indeed, another model for false memories is the activation-monitoring account (McDermott & Watson, 2001) that postulates two opposite processes. During the encoding phase, spontaneous and automatic activation of an associative network relating to the studied thematic list would occur. Conversely, at the recognition or recall phase, an efficient monitoring process would correctly associate the retrieval with the participant’s inherent thoughts and not with a previous presentation of the item within the studied list (Gallo, 2010; McDermott & Watson, 2001). Accordingly, the production of false memories could result from a greater activation of the critical item and/or from a defective source monitoring system. Accordingly, reducing the availability of attentional resources during encoding or retrieval using a divided attention procedure could mimic the effect of OSA in healthy participants (Sarhane & Daurat; 2019). However, this is somehow contradicted by evidence that some of the strongest false memory patterns reflect advanced cognitive handling, rather than deriving from impaired processing (Brainerd, Reyna, & Ceci, 2008; Brainerd, Reyna, & Zember, 2011). Indeed, whereas source monitoring errors were found more prevailing in cognitive immaturity conditions, e.g. in children (Brainerd & Reyna, 2005) and cognitively deficient adults (Law, Hawkins, & Craik 1998; Latour et al., 2014) notice the opposite pattern for false memory levels reported to be higher in adolescents than in children (e.g., Carneiro & Fernandez 2009), young adults than in adolescents (e.g., Lyons, Ghetti, & Cornoldi 2010), nondisabled than learning-disabled individuals (e.g., Weekes et al., 2007), non-autistic than autistic individuals (e.g., Hillier et al., 2007), and college students with higher versus lower SAT Reasoning Test scores (Brainerd et al., 2008). As compared to controls, patients with amnesia also exhibit reduced recognition scores both for correct and false-related items (Melo, Winocur, & Moscovitch, 1999; Ciaramelli et al., 2006, cited in De Brigard, 2014). This led some to name these « smart » false memories (Latour, Latour, & Brainerd, 2014), referring to the fact that they would result from a deep semantic processing (Brainer, Reyna, & Ceci, 2008).

Our results also showed that participants exhibit increased confidence in their memory recall (i.e., “remember” responses) after PAP treatment both for target and lure items. Together with increased recognition scores both for lures and targets, these results partly contradict the McCabe at al. (2009) proposal that decreased recall for learned items stems from an increase in false memories due to familiarity, or with the “Activation-Monitoring” assumption (McDermott & Watson, 2001) that a greater number of false memories would result from an inefficient source monitoring system. It has been proposed that patients with OSA have more difficulties consolidating the contextual and perceptual characteristics of the information (Barner et al., 2016). Patients with OSA would, therefore, be more likely to confuse different sources of information, leading to a higher production of false recognitions than controls (Sarhane et al., 2014; Sarhane & Daurat, 2017). However, our results show a concomitant increase in the production of “false” and “true” memories, which in the framework of these theories would paradoxically suggest a degradation in memory source after PAP treatment. Alternatively, if considering that efficient sleep benefits subsequent hippocampal activity and memory performance (Van Der Werf et al., 2009), and that the hippocampus plays an important role in binding information (Borders et al., 2017), it might also favor the induction of false memories using tasks such as the DRM paradigm, in which target and lure items are highly semantically related (Diekelmann, Born, & Wagner, 2010). Enhanced sleep quality (shown by diminished sleep fragmentation here) might thus facilitate the mechanisms by which themes are extracted from the learned lists, promoting automatic abstraction and generalization abilities (Diekelmann, Born & Wagner, 2010).

Accordingly, we found a correlation between PAP compliance over three months and acceptance score for semantically related lures, which may be interpreted as an increased tendency to abstract and retain the general theme following a PAP-related qualitative improvement of sleep. Prior studies already showed that the odds for normalization of verbal memory function after three months of CPAP treatment are related to compliance, being 7.9 times higher for optimal (average use > 6h per night) than poor (<2h per night) users (Zimmerman et al., 2006). In our population, average compliance rate was intermediate (5,1 ± 0.37 h per night). While some neurocognitive functions could benefit from PAP immediately after the beginning of the treatment, additional improvements in other neurocognitive domains might necessitate extended PAP treatment with good clinical compliance for patients with severe OSA (Lin et al., 2015). It was shown that one month of PAP treatment in patients with OSA leads to reduced daytime sleepiness and improved verbal episodic memory (Rosenzweig et al., 2016). This also corroborates our participants› subjective reports of a perceived improvement of memory skills and decreased sleepiness and fatigue at follow-up 3 months later.

Our study presents limitations that could be addressed in future research. First, the sample of participants at the three-month delay was reduced. This drop-out is representative of the known poor adherence and compliance to PAP treatment in part of the clinical population (Bakker et al., 2016). In this respect, highlighting rapid then enduring CPAP-related effects on cognitive processes would provide arguments to encourage the compliant use of PAP treatments. Second, this study was held in a sleep laboratory with recently diagnosed patients with moderate to severe OSA, who needed the rapid initiation of PAP treatment. It would have been ethically difficult to propose a sham PAP condition as a control, which leaves open the potential impact of motivational/placebo variables. Finally, one may wonder after the possibility of a repetition effect on performance, i.e. an improvement from one session to the other due to increased familiarity and practice on the DRM task. Although we did not run a control group to evaluate this potential repetition effect, we believe it unlikely as for the one hand, performance improvement only occurred from Session 1 (baseline) to Session 2 (PAP treatment) but did not further improve at Session 3 (3-months follow-up), and on the other hand, another study did not find any between-session improvement or strategic effects after repeated administration of the DRM test using different learning lists (Dehon, submitted).

## Conclusion

To sum up, this study provides evidence for an immediate and persistent CPAP treatment-related improvement in both gist and item-specific verbal memory in OSA patients. This demonstrates a direct benefit of CPAP treatment on learning and memory generalization mechanisms and extent prior findings showing beneficial effects of CPAP treatment in various domains, from daytime functioning to higher-order cognitive functions.

## Additional File

This article has an online data supplement available on OSF database at *https://osf.io/ra27m/*.
